# Galectin-9 contributes to the pathogenesis of atopic dermatitis *via* T cell immunoglobulin mucin-3

**DOI:** 10.3389/fimmu.2022.952338

**Published:** 2022-07-22

**Authors:** Wenxing Su, Ji Zhang, Shun Yang, Minhui Tang, Yu Shen, Cuiping Liu, Jiang Ji, Marcus Maurer, Qingqing Jiao

**Affiliations:** ^1^ Department of Dermatology, The Second Affiliated Hospital of Soochow University, Su Zhou, China; ^2^ Department of Dermatology, The First Affiliated Hospital of Soochow University, Suzhou, China; ^3^ Department of Plastic and Burn Surgery, The Second Affiliated Hospital of Chengdu Medical College (China National Nuclear Corporation 416 Hospital), Chengdu, China; ^4^ Jiangsu Institute of Clinical Immunology and Jiangsu Key Laboratory of Clinical Immunology, The First Affiliated Hospital of Soochow University, Suzhou, China; ^5^ Institute of Allergology, Charité – Universitätsmedizin Berlin, Corporate Member of Freie Universität Berlin and Humboldt-Universität zu Berlin, Berlin, Germany; ^6^ Allergology and Immunology, Fraunhofer Institute for Translational Medicine and Pharmacology ITMP, Berlin, Germany

**Keywords:** T cell immunoglobulin- and mucin-domain-containing molecules-3 (TIM-3), Galectin-9 (Gal-9), T_H_1 cells, T_H_2 cells, T_H_17 cells, T_H_22 cells, atopic dermatitis

## Abstract

**Background:**

Atopic dermatitis (AD), a common type 2 inflammatory disease, is driven by T helper (T_H_) 2/T_H_22polarization and cytokines.Galectin-9 (Gal-9), *via* its receptor T cell immunoglobulin- and mucin-domain-containing molecule-3 (TIM-3), can promote T_H_2/T_H_22 immunity. The relevance of this in AD is largely unclear.

**Objectives:**

To characterize the role of TIM-3 and Gal-9 in the pathogenesis of AD and underlying mechanisms.

**Methods:**

We assessed the expression of Gal-9 and TIM-3 in 30 AD patients, to compare them with those of 30 healthy controls (HC) and to explore possible links with disease features including AD activity (SCORAD), IgE levels, and circulating eosinophils and B cells. We also determined the effects of Gal-9 on T cells from the AD patients.

**Results:**

Our AD patients had markedly higher levels of serum Gal-9 and circulating TIM-3-expressing T_H_1 and T_H_17 cells than HC. Gal-9 and TIM-3 were linked to high disease activity, IgE levels, and circulating eosinophils and/or B cells. The rates of circulating TIM-3-positive CD4^+^ cells were positively correlated with rates of T_H_2/T_H_22 cells and negatively correlated with rates of T_H_1/T_H_17 cells. Gal-9 inhibited the proliferation and induced the apoptosis of T cells in patients with AD, especially in those with severe AD.

**Conclusion:**

Our findings suggest thatGal-9, *via* TIM-3, contributes to the pathogenesis of AD by augmenting T_H_2/T_H_22 polarization through the downregulation of T_H_1/T_H_17immunity. This makes Gal-9 and TIM-3 interesting to explore further, as possible drivers of disease and targets of novel AD treatment.

## Introduction

Atopic dermatitis (AD) is a common T-cell mediated skin inflammatory disease, with abnormal activation of several subpopulations of T helper (T_H_)cells (1–3). We and others have shown that AD, in many patients, is characterized by excess T_H_2/T_H_22 cell activity (4–8). The increased production of T_H_2 cytokines such as interleukin (IL)-4 and IL-13 initiates a complex immune cascade that includes the generation of allergen-specific IgE-producing B cells and eosinophil migration to AD skin lesions (9–14), two hallmark features of AD. Furthermore, T_H_2 and T_H_22 cytokines inhibit skin barrier protein-encoding genes such as filaggrin, loricrin, and involucrin (15) and the production of antimicrobial peptides, both of which are held to contribute to the increased susceptibility to skin infections in patients with AD (16). The key role of T_H_2/T_H_22 cytokines in the pathogenesis of AD is supported by the efficacy of treatment with the anti-IL-4 receptor antibody dupilumab and an anti-IL-22 antibody (ILV-094) (17–19). As of now, it is largely unclear what drives T_H_2/T_H_22 skewing in AD.

Galectin-9 (Gal-9) is a tandem-repeat type galectin with two carbohydrate-recognition domains, and it was first identified as an eosinophil chemoattractant and activation factor (20, 21). It is universally expressed in a wide range of immune and non-immune cells and is known to regulate different biological functions, such as cell adhesion, differentiation, aggregation, and cell death (22). Galectin-9 is a versatile immunomodulator that has recently been shown to be associated with the pathogenesis of AD. For example, the skin of AD patients exhibits increased levels of Gal-9, especially in the epidermis, and increased numbers of Gal-9 positive eosinophils and mast cells (23). Blood levels of Gal-9, in patients with AD, were reported to be significantly higher than in healthy controls (HC) and correlated with disease activity (24).

Gal-9 exerts its biological functions *via* multiple receptors, including CD44 and T-cell immunoglobulin and mucin containing-protein 3 (TIM-3). TIM-3 is expressed by several populations of immune cells including terminally differentiated T_H_1, T_H_17, and Tc1 lymphocytes as well as NK, monocytes, and myeloid cells, whereas T_H_2/T_H_22 cells do not express TIM-3 (25). Gal-9 signaling *via* TIM-3 is held to modulate immune responses and diseases. For example, we have previously shown upregulation of Gal-9 and TIM-3 in the serum and peripheral blood mononuclear cells of patients with systemic lupus erythematosus (SLE), and this was closely related to disease activity (26). Gal-9, *via* TIM-3, induces apoptosis in T_H_1 and T_H_17 cells (27, 28), is involved in tolerance induction and T cell exhaustion (25, 27, 29, 30), and downregulates T_H_1/T_H_17-biased immune responses resulting in T_H_2 polarization. Whether or not TIM-3 plays a role in AD is currently unknown.

To address this question, we investigated patients with AD and HC for their Gal-9 serum levels and rates of circulating TIM-3-positive cells, we characterized the clinical relevance of Gal-9 and TIM-3 in AD, and we explored potential mechanisms that underlie their role in the pathogenesis of AD. 

## Materials and methods

### Study conduct, patients, and control subjects

Ethical approval from the Ethics Committee of The First Affiliated Hospital of Soochow University (Suzhou, China, No. 2014809026) was obtained prior to the study. All patients provided written informed consent in accordance with the Helsinki Declaration of the World Medical Association. AD was diagnosed in accordance with the criteria of Hanifin and Rajka and disease severity was evaluated using the SCORing Atopic Dermatitis index (SCORAD), with 0–24, 25-50, and >50 points reflecting mild, moderate, and severe AD, respectively (31). At the time of the study and for one month prior, none of the patients were treated with systemic steroids or other immunosuppressant treatments, or potent topical steroids, or topical corticosteroids as well as other medications (e.g. antibiotics, light therapy ect.). Patients with other allergic conditions, e.g., pollen allergy, food allergy, or allergic asthma, et al. were excluded. Age-matched healthy blood donors were recruited as controls, all of whom were without any allergic conditions (n =30, female: 19; mean age: 10.4 ± 4.7 years).Pediatric allergy specialists and trained field technicians performed the physical examinations, SCORAD score assessments, and collected blood samples.

Laboratory investigation including blood routine examination and IgE levels. Total and specific IgE levels measured at the central laboratory (Central Labor, The Second Affiliated Hospital of Soochow University) using Immuno CAP System (Phadia Laboratory Systems, Thermo Fisher Scientific Inc, Uppsala, Sweden).

### Peripheral blood mononuclear cell purification

PBMCs were immediately isolated and purified from drawn blood as previously published (32). Briefly, PBMCs were isolated from heparinized venous blood on Ficoll-Hypaque gradients (Pharmacia, Uppsala, Sweden) and re-suspended in Roswell Park Memorial Institute (RPMI) 1640 medium supplemented with gentamicin (40μg/mL) and 10% pooled type AB normal human serum (Sigma-Aldrich).

### Flow cytometric analyses

T cells were stained with fluorescent-labeled monoclonal antibodies against CD-4-FITC (300538, Clone: RPA-T4, Biolegend), TIM-3-PE (345006, Clone: F38-2E2, Biolegend), IFN-γ-APC (502512, Clone: 4S.B3, Biolegend), IL-17A-APC (512334, Clone: BL168, Biolegend), IL-4-APC (500812, Clone: MP4-25D2, Biolegend), and IL-22-APC (366706, Clone: 2G12A41, Biolegend). Intracellular staining was performed as follows: Surface staining was performed for 20 minutes with CD-4-FITC and/or TIM-3-PE antibodies on ice. Cells were washed and resuspended in fixation/permeabilization solution (420801/421002, Biolegend) and stained with IFN-γ-APC, IL-17A-APC, IL-4-APC, and IL-22-APC. B cells were stained with CD19-PerCP (392510, Clone: 4G7, Biolegend). Armenian hamster IgG (400908, Clone: HTK88, Biolegend), mouse IgG1 (400108, Clone: RTK2071, Biolegend), mouse IgG2a (400246, Clone: MOPC-173, Biolegend), and mouse IgG 2b (400314, Clone: MPC-11, Biolegend) were used as isotype controls. Cells were analyzed with a Coulter Epics XL Flow cytometer (Beckman) and a Coulter FC 500 ANALYZER (Beckman Coulter); the relevant data were obtained and analyzed using FlowJo software, version 7.6.

### Gal-9, PBMC proliferation, apoptosis, and cytokine production analysis

For analysis of the Gal-9 level, serum was obtained by centrifuging peripheral blood samples (PBs) from patients with AD; the level of expression of Gal-9 in serum was detected using ELISA kits (AMS Biotechnology, UK), and the PBMC were separated by density gradient centrifugation. Cells from the interphase were collected and washed twice with Dulbecco’s PBS. For analysis of proliferation, apoptosis, cytokine production, freshly isolated PBMCs (1 × 10^5^ cells/well) were cultured in RPMI 1640 medium (Gibco, USA) containing 10% human AB serum (Gibco) with Recombinant Gal-9 (0.5µg/mL, 1µg/mL, 2µg/mL, and 4µg/mL, ICA309Bo01, LMAI Bio) and LEAFTM Purified Anti-Human CD3 Antibody (100 ng/mL, BioLegend) in 96-well plates for 72 hours, respectively. For analysis of cell proliferation, cell viability was determined using a Cell Counting Kit-8 (CCK-8) assay kit (Beyotime Institute of Biotechnology, Beijing, China). Cells were stained with annexin V-FITC and PI to detect early apoptotic cells (annexin V positive, PI negative) and late apoptotic cells (annexin V positive, PI positive) by flow cytometry (BD PharMingen).

### Statistical analysis

Statistical analysis and Figures were performed or made using GraphPad Prism 5 (GraphPad Software, La Jolla, CA, USA), respectively. The distribution of numerical variables were analyzed with the Kolmogorov-Smirnov test. Nonparametric tests were used for not normally distributed data. The relation between TIM-3 or Gal-9 expression level and clinical and laboratory characteristics was examined by Spearman’s or Pearson’s correlation coefficient rank test. Comparison analyses between the groups were carried out using the χ^2^ test, the Mann-Whitney U test, and the Friedman test. A P-value ≤ 0.05 was considered statistically significant.

## Results

### Blood levels of Gal-9 and TIM-3-positive T cells are markedly increased in patients with AD

**Figure 1:**
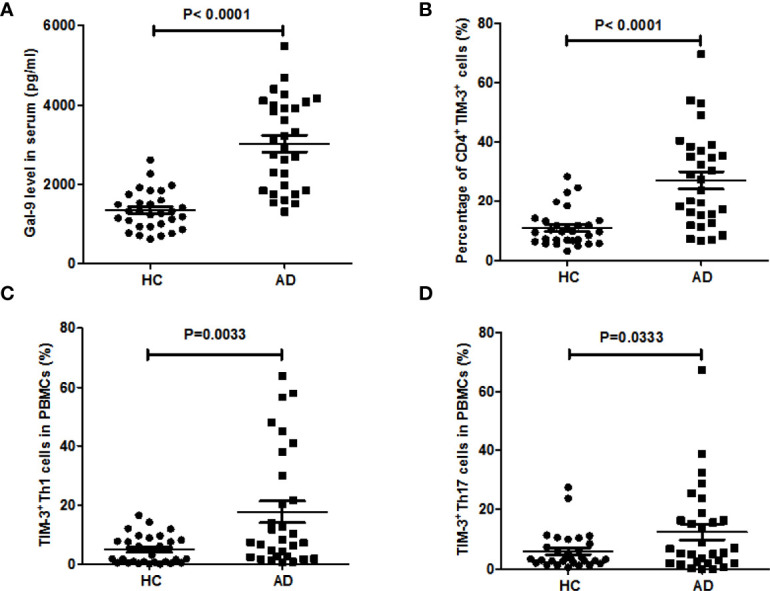
Blood levels of Gal-9 and TIM-3-positive T cells are markedly increased in patients with AD. **(A)** Serum Gal-9 levels of patients with atopic dermatitis involved in this study (AD, n = 30), as compared to healthy controls (HC, n = 30), **(B)** the percentage of CD4^+^TIM-3^+^ T cells in whole blood, the percentage of **(C)** TIM-3^+^T_H_1 cells and **(D)** TIM-3^+^T_H_17 cells in PBMCs of the above patients with AD compared to HC. These results are presented as means ± SEM.

A total of 30 AD patients (female: 20; mean age: 11.1 ± 6.0 years; aged 1-3 years, n=2; aged 3-5 years, n=5; aged 5-12 years, n = 10; aged 12-18 years, n=10; aged 18-20, n=3) were included after informed consent. As was previously reported, patients with AD had markedly higher serum levels of Gal-9, as compared to HC (3,030 ± 208 *vs* 1,330 ± 90 pg/ml, P<0.0001, **Figure 1A**). In addition, AD patients showed significantly higher rates of TIM-3-positive (TIM-3^+^) circulating CD4^+^ T cells (27.2 ± 2.9%*vs* 11± 1.1%, P<0.0001, **Figure 1B** and **S1**).In CD4^+^IFN-γ^+^ cells(T_H_1 cells), rates of TIM-3 expression were more than 3-fold higher in AD patients (17.7 ± 3.6% *vs* 4.9± 0.9%, P=0.003, **Figure 1C**, and **S2**), and rates in CD4^+^ IL-17A^+^ cells(T_H_17 cells) were twice as high, as compared to HC (12.5± 2.7%*vs* 6.1± 1.2%, P=0.033, **Figure 1D** and **S3**) (**Table 1**).

**Table 1:** Characteristics of patients with atopic dermatitis in the present study.

Patient		Age at onset		Total serum	Absolute eosinophil count	Blood CD19^+^ B cells	
**no.**	**Sex**	**(years)**	**SCORAD**	**IgE (U/ml)**	**(/nl) (0,02-0,52)**	**(%)**	**Allergens**
**1**	F	6	17.20	878	1.22	8.49	*D.pteronyssinus*
**2**	F	5	19.50	86.12	0.29	6.17	/
**3**	F	6	20.10	2979	3.76	9.25	/
**4**	F	5	23.70	199.40	1.87	15.60	Cat and Dog dander
**5**	F	11	16.20	2026	0.28	14.90	unknown
**6**	F	5	15.10	617	0.67	6.71	unknown
**7**	M	19	6.80	1586	1.73	13.80	Dog dander and Birch pollen
**8**	F	18	11.80	2773	0.02	12.10	*C. albicans*
**9**	F	20	12.60	3300	1.50	13.50	/
**10**	M	10	27.50	1084	3.84	11.80	/
**11**	F	2	29.20	710	1.90	13.20	/
**12**	F	13	30.50	2783	4.70	11.90	Birch pollen
**13**	M	6	32.50	2173	5.01	16.00	/
**14**	M	8	34.80	580	1.80	15.00	/
**15**	M	14	35.40	440	5.10	11.40	Peanut and Shrimp
**16**	M	16	37.20	2069	3.60	7.28	Cat dander
**17**	F	18	38.50	1099	1.80	13.40	Mugwort pollen
**18**	M	15	69.50	1945	0.10	9.67	Timothy pollen
**19**	F	16	70.20	2118	1.19	11.20	/
**20**	F	4	75.10	738	3.73	6.52	/
**21**	F	13	78.20	3769	2.03	17.20	*D.pteronyssinus*
**22**	M	18	53.00	1737	1.01	7.60	/
**23**	M	20	54.00	1165	3.04	9.88	Egg white and Cow’s milk
**24**	M	19	56.50	1198	4.07	15.40	unknown
**25**	F	6	61.30	2945	0.12	6.39	unknown
**26**	F	8	68.40	3227	0.10	10.60	*D.pteronyssinus*
**27**	F	17	39.20	1175	0.26	7.48	/
**28**	F	1	40.50	2284	0.13	11.60	/
**29**	F	8	45.60	2889	0.30	13.60	/
**30**	F	7	49.00	2076	0.04	12.90	unknown

F, female; M, male; /, no testing for sensitization to allergens was performed. SCORAD, SCORing Atopic Dermatitis. Determination of suspected allergenswas performed by fluorescence enzyme immunoassay usingImmunoCAP System^®^ (Sigma-Aldrich, Deisenhofen, Germany).

**Figure 2:**
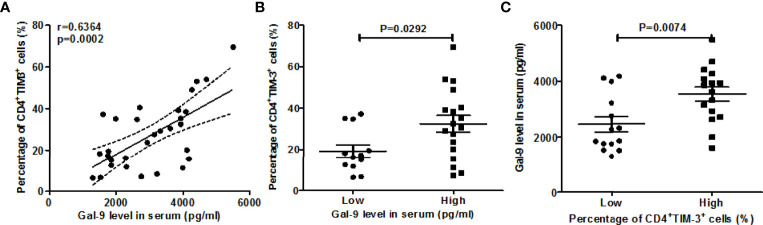
Correlation between Gal-9 levels and rates of TIM-3+CD4+ T cells. **(A)** Association between the percentage of TIM-3+CD4+ T cells and serum Gal-9 levels. For comparisons between groups, we divided the data based on Gal-9 level as low (<2659.13pg/ml), high (≥2659.13pg/m) and the frequency of TIM-3+ cells on CD4+ T cells as low (<21.9%), high (≥21.9%), respectively. The above cut-off values were 2 times the mean of HC test results. **(B, C)** The association of the serum Gal-9 level and the percentage of TIM-3+CD4+ T cells.

Furthermore, serum Gal-9 levels were strongly and positively correlated with rates of circulating TIM-3^+^CD4^+^ T cells (r=0.6364, P=0.0002, **Figure 2A**). AD patients with high serum levels of Gal-9 had markedly higher rates of TIM-3^+^CD4^+^ T cells as compared toAD patients with low serum levels ofGal-9 (32.5 ± 4%*vs* 19.3± 3.9%, P=0.029, **Figure 2B**).Vice versa, patients with high rates of TIM-3^+^CD4^+^ T cells had markedly higher serum levels of Gal-9 than patients with low rates (3,532 ± 253 *vs* 2,456 ± 273 pg/ml, P=0.0074, **Figure 2C**).

### In AD, high rates of circulating TIM-3^+^T cells are linked to high disease activity, IgE levels, and circulating eosinophils and B cells

**Figure 3:**
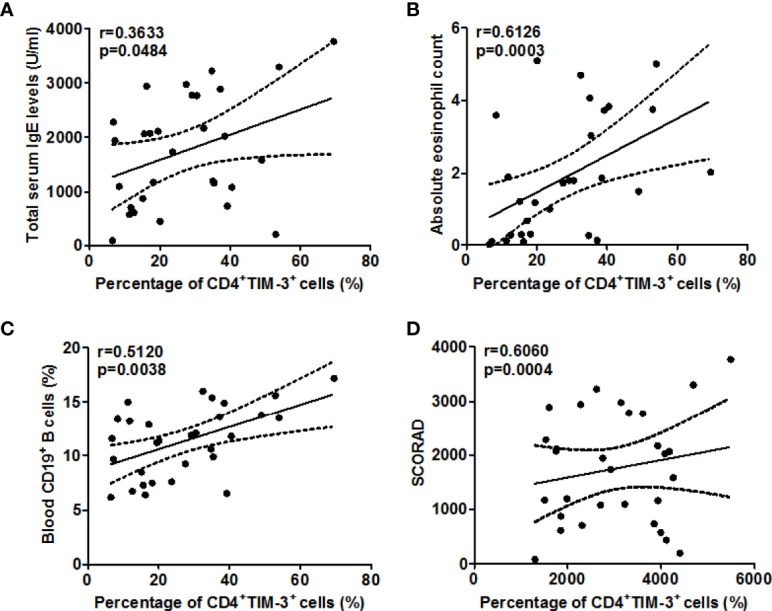
Association between the percentage of TIM-3^+^CD4^+^ T cells and total serum IgE **(A)**, circulating eosinophils **(B)**, blood CD19^+^ B cells **(C)**, and disease activity as assessed by SCORAD **(D)** in patients with AD.

**Figure 4:**
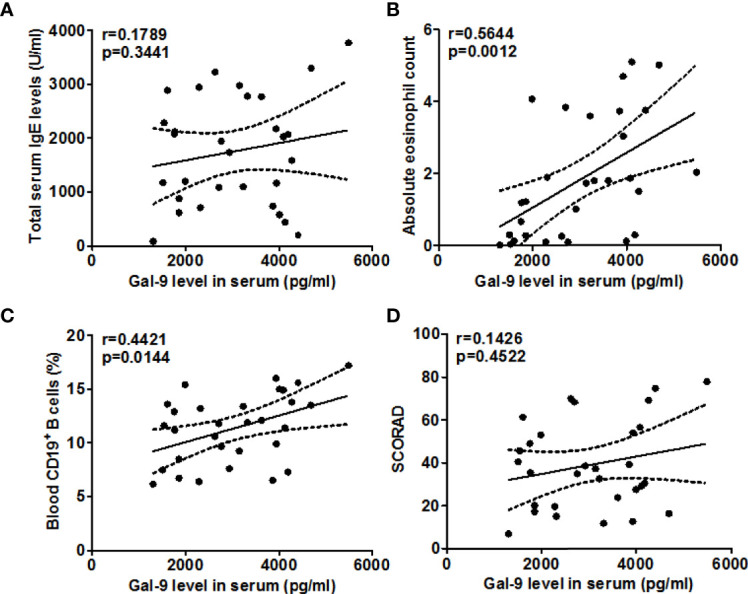
Association between the serum Gal-9 levels and total IgE **(A)**, eosinophils **(B)**, CD19^+^ B cells **(C)**, and SCORAD **(D)** in patients with AD.

When we assessed these findings for their clinical relevance, increased circulating TIM-3^+^CD4^+^T cell populations in our AD patients were linked to higher disease activity, i.e. SCORAD values (r =0.6060, P=0.0004, **Figure 3D**), higher serum levels of total IgE (r =0.3633, P=0.048, **Figure 3A**), as well as higher number of circulating blood eosinophils (r =0.6126, P=0.0003, **Figure 3B**) and CD19^+^B cells (r =0.5120, P=0.0038, **Figure 3C**). Gal-9 serum levels showed similar links, albeit less pronounced (**Figure 4**), suggesting that TIM-3 and Gal-9 contribute to the course and pathogenesis of AD.

### In AD, rates of circulating TIM-3-positive CD4^+^ cells are positively correlated with rates of T_H_2/T_H_22 cells and negatively correlated with rates of T_H_1/T_H_17 cells

**Figure 5:**
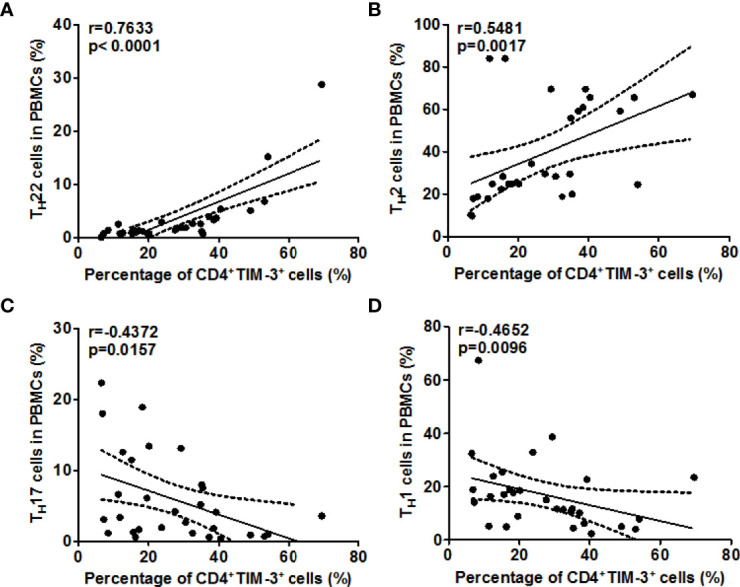
The correlation between TIM-3 levels and T_H_2/T_H_22 as well as T_H_1/T_H_17 cell ratios in AD. Association between the percentage of TIM-3^+^CD4^+^ T cells and the frequency of T_H_1 cells **(A)**, T_H_2 cells **(B)**, T_H_17 cells **(C)**, and T_H_1 cells **(D)** in patients with AD.

**Figure 6:**
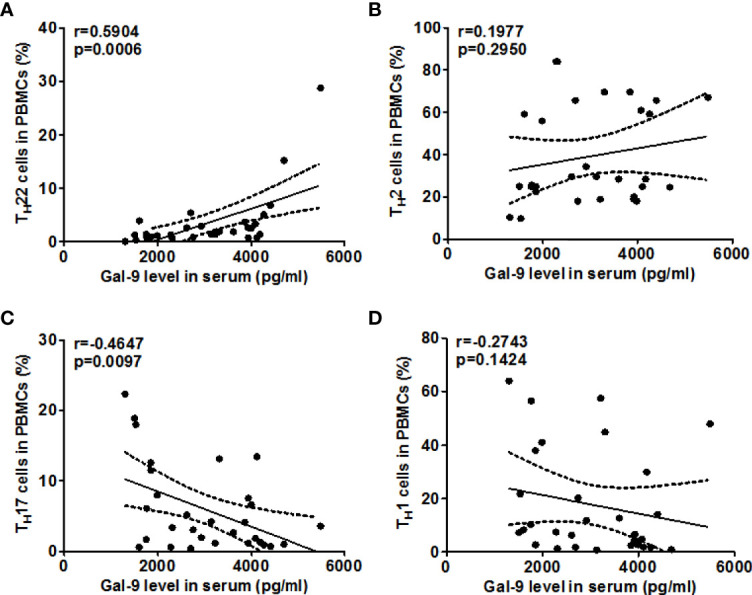
The correlation between Gal-9 levels and T_H_2/T_H_22 as well as T_H_1/T_H_17 cell ratios in AD. Association of Gal-9 levels and the percentage of TIM-3^+^CD4^+^ T cells and frequency of T_H_1 cells **(A)**, T_H_2 cells **(B)**, T_H_17 cells **(C)**, and T_H_22 cells **(D)** in patients with AD.

Next, we explored the role of TIM-3 and possible underlying mechanisms in AD. High rates of TIM-3^+^CD4^+^T cells in the blood of AD patients were strongly linked with high rates of T_H_22 cells (r=0.7633, P<0.0001, **Figure 5A**), and, in addition, with those of T_H_2 cells (r =0.5481, P <0.01, **Figure 5B**). In contrast, the rates of TIM-3^+^CD4^+^ T cells in the blood of our AD patients were negatively correlated, albeit weakly, with those of T_H_17 cells (r =-0.4372, P <0.05, **Figure 5C**), and, additionally, with those of T_H_1 cells (r =-0.4652, P <0.01, **Figure 5D**). Serum levels of Gal-9, in our AD patients, were also positively and negatively correlated with circulating T_H_22 cells (r=0.5904, P <0.001, **Figure 6A**) and T_H_17 cells (r =-0.4647, P <0.01, **Figure 6C**), respectively, suggesting that Gal-9, *via* TIM-3, downregulates T_H_1/T_H_17-immunity and drives T_H_2/T_H_22 polarization. However, there were no significant correlations between Gal-9 serum levels and circulating TH2 cells (**Figure 6B**) and TH1 cells (**Figure 6D**).

### Gal-9 inhibition of T cell proliferation and induction of T cell apoptosis are linked to AD severity

**Figure 7:**
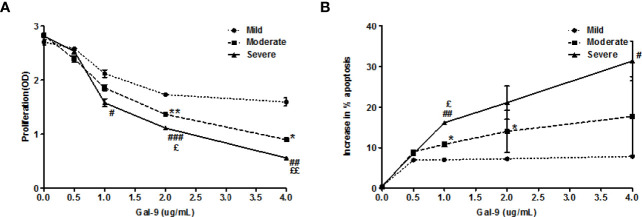
Gal-9 inhibits the proliferation and induces apoptosis in PBMCs from AD patients. **(A)** CCK-8 assay and **(B)** Apoptosis assay of PBMCs isolated from AD patients (Mild AD, n=11; Moderate AD, n=9; Severe AD, n=10) under graded doses of Gal-9 (0, 1.5, 5, 15, and 50 nM) and anti-CD3(100ng/ml) for 72 h, respectively. These results are presented as means ± SEM. *P <0.05, **P <0.01, ***P <0.001 Mild AD *vs*. Moderate AD; #P < 0.05, ##P < 0.01, ###P < 0.001 Mild AD *vs*. Severe AD. ζP < 0.05, ζζP < 0.01, ζζζP < 0.001 Moderate AD *vs*. Severe AD.

Finally, we characterized the effects of TIM-3 activation of T cells by Gal-9 in AD and their clinical relevance. To this end, we stimulated circulating T cells in PBMC samples of patients with mild, moderate, or severe AD with Gal-9 and anti-CD3 and then assessed their proliferation and apoptosis. Gal-9 dose-dependently inhibited proliferation (**Figure 7A**) and induced apoptosis (**Figure 7B**) in PBMCs of AD patients. The inhibition of proliferation and induction of apoptosis by Gal-9 were highest in PBMCs of patients with severe AD and lowest in patients with mild AD (**Figures 7A, B**), linking Gal-9 effects on T cells to AD disease severity.

## Discussion

This study tiesGal-9 and its receptor, TIM-3, to the pathogenesis of AD. Both are upregulated in patients in AD and linked to disease features and activity. Our findings support the notion that Gal-9, *via* TIM-3, augments T_H_2/T_H_22 polarization and down regulates T_H_1/T_H_17immunityvia effects on CD4^+^ T_H_1 and T_H_17 cells.

That Gal-9 levels are elevated in AD is not a new finding (23, 33). In contrast, what our study shows for the first time, is that levels of CD4^+^ T cells that express the Gal-9 receptor TIM-3 are also markedly increased in patients with AD. TIM-3 is specifically expressed in T_H_1 and T_H_17 cells, but not in T_H_2 (33), and our AD patients showed triple and double the rate of TIM-3-expressing T_H_1 and T_H_17 cells, respectively, as compared to HC. These findings go against those reported by Kanai and coworkers, who reported numbers of TIM-3-expressing CD4^+^T cells to be similar in 9 AD patients as compared to HC (34). Possible explanations for this discrepancy include differences in patient populations, i.e. young Han Chinese patients in our study *vs* middle-aged Japanese patients, and the small number of patients studied. In addition to age, factors such as gender, genetics, and environmental factors also will influence the immunological profile of patients with AD (35).

Why are Gal-9 levels and rates of TIM-3^+^CD4^+^ T cells both upregulated in AD? Our study does and cannot answer this question and was not meant to. Further studies are needed to identify the underlying mechanisms. At least four scenarios could be relevant. First, elevated Gal-9 could increase the rate of TIM-3^+^CD4^+^ T cells. Second, TIM-3^+^CD4^+^ T cells could drive Gal-9 levels. Third, Gal-9 and TIM-3 expression may be upregulated by independent mechanisms. Fourth, increased Gal-9 and TIM-3 expression may be driven by the same signals. The first scenario is unlikely since Gal-9 inhibits the proliferation and induces apoptosis of TIM-3^+^ cells, as previously reported (36)and demonstrated by our findings in AD. That TIM-3^+^cells produce or induce the production of Gal-9, i.e. scenario two, is also unlikely. CD4^+^T cells have been reported to produce Gal-9 (37, 38), but other cells such as keratinocytes and mast cells are probably much more relevant sources of Gal-9 in AD (24). As for the third and fourth options, the fact that Gal-9 levels and rates of TIM-3^+^CD4^+^T cells are strongly correlated suggests that the mechanisms that drive the elevation of both are shared, at least in part, rather than independent. Since both are not only correlated with each other, but also linked to disease activity and, to a lesser extent, AD features such as IgE and blood eosinophils and B cells, it appears likely that what drives the increase in Gal-9 levels and rates of TIM-3^+^CD4^+^T cells in AD is AD itself. Thus, Gal-9 and TIM-3 may act as amplifiers of AD pathogenesis. This notion is supported by the observation that effective treatment of AD can result in the decline of Gal-9 levels.

Our results clearly show that, regardless of the cause, high rates of circulating TIM-3^+^T cells are linked to high AD disease activity, IgE levels, numbers of circulating eosinophils and B cells, as well as high rates of T_H_2/T_H_22 cells and low rates of T_H_1/T_H_17 cells. This was also so for Gal-9, albeit less pronounced. What explains this, at least in part, is that Gal-9, in our AD patients, inhibits T cell proliferation and induces T cell apoptosis and that both effects are linked to AD severity. The vicious feedback loop suggested by our results looks like this: High levels of Gal-9 and high levels of TIM-3 expressing T_H_1/T_H_17 cells make for strong inhibition of T_H_1/T_H_17 immunity and for T_H_2/T_H_22 polarization, which in turn comes with high levels of disease activity and inflammatory signals that may drive further Gal-9 and TIM-3 expressions.

As two target glycoproteins of Gal-9 have been identified, TIM-3 and CD44. Whether Gal-9 downregulates T_H_1/T_H_17 immunity *via* TIM-3 in AD? First, we observed that both Gal-9 level and the rate of TIM-3^+^CD4^+^ T cells are elevated in AD patients. Second, Gal-9 levels and rates of TIM-3^+^CD4^+^T cells are strongly correlated in our patients with AD. Third, in our AD patients, Gal-9 significantly inhibited T cell proliferation and induced T cell apoptosis. These results indicate that Gal-9 might *via* TIM-3 contributes to the inhibition of T_H_1/T_H_17 activation in AD. However, further experimental evidence is still needed, such as TIM-3 block experiment. And additional experiments with galectin inhibitors also need to be performed to clarify the specific mechanism of Gal-9-mediated suppression in AD.

Our study has several strengths and a few limitations. As for the former, for example, we assessed Gal-9 and TIM-3 in a sizeable and well-characterized patient population, together with clinical and other molecular markers. A major limitation of our study is its monocentric approach, which calls for confirmation of our results in a broader and more heterogeneous group of patients. A minor limitation of our study is that we only investigates the expression of Gal-9 and TIM-3 in blood samples. This is mainly due to blood samples are relatively easy to obtain, and blood source indicators have the potential to be developed into biomarkers in the later stage for AD. As skin biopsy is not a routine test for patients with AD. Besides, two studies have been reported that, increased Gal-9 expression in the skin lesions of AD patients (23, 24). Whereas, comparative studies on Gal-9 and TIM-3 expression in peripheral blood and lesions of AD are still needed.

**Figure 8:**
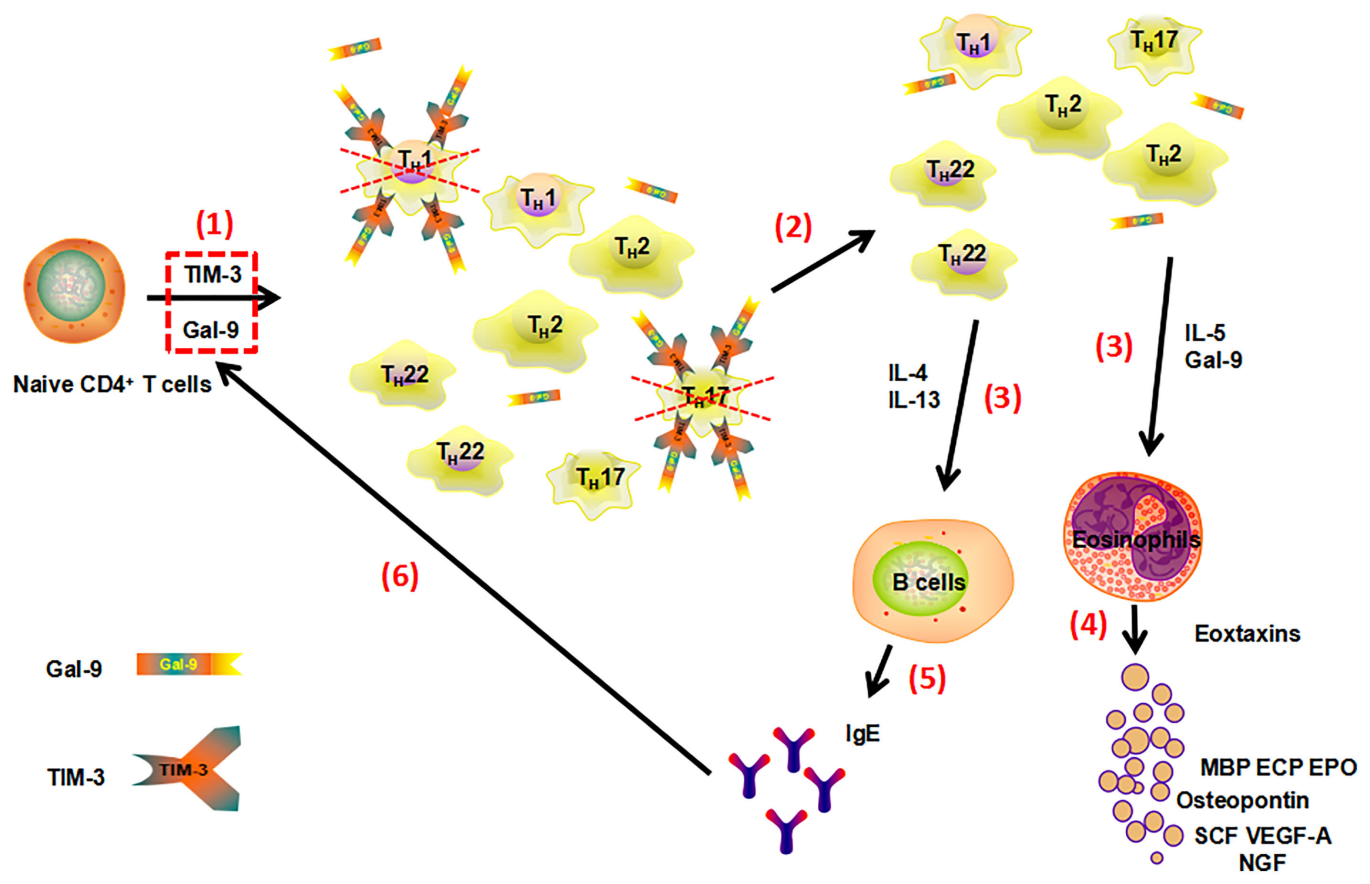
Proposed model of the role of Gal-9 and TIM-3 in the pathogenesis of AD. Gal-9, *via* TIM-3 expressed by T_H_1/T_H_17 cells, downregulates their numbers, by inhibiting proliferation and the induction of apoptosis (1). The reduction of T_H_1/T_H_17 immunity leads to T_H_2/T_H_22 polarization (2). Increased T_H_2/T_H_22 immunity and cytokines drive type 2 inflammation and disease activity (3) with higher numbers of eosinophils(4) and B cell class switching to IgE and elevated IgE levels (5). This, in turn, may drive further upregulation of Gal-9 and TIM-3 expression (6). MBP (Major basic protein), ECP (Eosinophil cationic protein), EPO (Eosinophilperoxidase), SCF (Stem cell factor), VEGF-A (Vascular endothelial growth factor-A), NGF (Nerve growth factor).

Taken together, as summarized in **Figure 8**, upregulation of TIM-3/Gal-9 interaction, in AD, comes with downregulation of T_H_1/T_H_17 responses and more pronounced T_H_2/T_H_22 immunity. Our data suggest that the TIM-3/Gal-9 pathway may play an important role in the pathogenesis of AD, given that levels of TIM-3/Gal-9 are closely associated with disease activity, total serum IgE levels as well as blood eosinophil and B cell count. Further research is needed to clarify the molecular mechanisms that drive increased TIM-3/Gal-9 expression of T_H_1/T_H_17 cells in AD. In addition, future studies should aim to characterize TIM-3/Gal-9 expression on Tc1, NK, and myeloid cells as well as their levels in skin lesions of patients in AD.

## What is already known about this topic?

- Atopic dermatitis (AD) is driven by T_H_2/T_H_22 polarization and cytokines.
- Galectin-9 (Gal-9) can promote T_H_2/T_H_22 immunity, *via* its receptor T cell immunoglobulin- and mucin-domain-containing molecule-3 (TIM-3).

## What does this study add?

- Gal-9 and TIM-3 are markedly upregulated in AD and linked to disease features.
- Gal-9 and TIM-3 levels are positively correlated with rates of T_H_2/T_H_22 cells and negatively correlated with rates of T_H_1/T_H_17 cells.
- TIM-3/Gal-9 inhibits the proliferation and induces apoptosis in AD T cells, and both effects are linked to disease severity.

## Data availability statement

The original contributions presented in the study are included in the article/**Supplementary material**. Further inquiries can be directed to the corresponding author/s.

## Ethics statement

This study was reviewed and approved by The Ethics Committee of The First Affiliated Hospital of Soochow University (Suzhou, China, No. 2014809026). Written informed consent to participate in this study was provided by the participants’ legal guardian/next of kin.

## Author contributions

WS, JZ, SY, YS, and CL performed experiments and analyzed the data. MT and JJ recruited patients. QJ, JJ, and MM analyzed data and wrote the manuscript. MM contributed to the critical revision of the manuscript. All authors contributed to the article and approved the submitted version.

## Funding

This work was supported by grants from the National Natural Science Foundation of China (No. 82073434), the Medical Scientific Research Project of Jiangsu Health Commission 2020 (Z2020017), Suzhou Minsheng Technology-Medical and Health Application Foundation (SYS2020135), The Opening Project of State Key Laboratory of Radiation Medicine and Protection (GZK1201912), and The Assistance Project of Suzhou Key Medical Discipline (SZFCXK202118).

## Conflict of interest

Author WS was employed by China National Nuclear Corporation 416 Hospital.

The remaining authors declare that the research was conducted in the absence of any commercial or financial relationships that could be construed as a potential conflict of interest.

## Publisher’s note

All claims expressed in this article are solely those of the authors and do not necessarily represent those of their affiliated organizations, or those of the publisher, the editors and the reviewers. Any product that may be evaluated in this article, or claim that may be made by its manufacturer, is not guaranteed or endorsed by the publisher.

## Supplementary material

Supplementary Figure 1 Representative flow cytometry analysis of TIM-3 expression on CD4^+^T cells in whole blood of patients with AD and HC. Cells were initially gated on CD4^+^ T cells. Subsequently, the frequency of TIM-3^+^ cells CD4^+^ T cells was analyzed by flow cytometry in the whole blood of AD patients and HC, respectively.

Supplementary Figure 2 Representative flow cytometry analysis of TIM-3 expression on CD4^+^IFN-γ^+^ T cells in PBMCs from AD patients and HC. Cells were initially gated on CD4^+^ T cells. Then IFN-γ^+^cells were selected out of the gated CD4^+^ T cells. Subsequently, the frequency of TIM-3^+^ cells on CD4^+^IFN-γ^+^ T cells was analyzed by flow cytometry in PBMCs from AD patients and HC, respectively.

Supplementary Figure 3 Representative flow cytometry analysis of TIM-3 expression on CD4^+^IL-17A^+^ T cells in PBMCs from AD patients and HC. The cells were initially gated on CD4^+^ T cells. Then IL-17A^+^ cells were selected out of the gated CD4^+^ T cells. Subsequently, the frequency of TIM-3^+^ cells on CD4^+^IL-17A^+^ T cells was analyzed by flow cytometry in PBMCs from AD patients and HC, respectively.

Supplementary Figure 4 Representative flow cytometry analysis of T_H_22 and T_H_22 in PBMCs from AD patients. **(A)** T_H_22 cells: Cells were initially gated on CD4^+^ T cells. Then IL-22^+^cells were selected out of the gated CD4^+^ T cells. **(B)** T_H_2: Cells were initially gated on CD4^+^ T cells. Then IL-4^+^cells were selected out of the gated CD4^+^ T cells.

## Abbreviations

AD, Atopic dermatitis; T_H_2, T helper T_H_ 2; TIM-3, T cell immunoglobulin- and mucin-domain-containing molecules-3; Gal-9, Galectin-9; HC, Healthy controls; TIM, T cell immunoglobulin mucin; PBMCs, Peripheral Blood Mononuclear Cells; AV/PI, Annexin V/propidiumiodide.
