# Supplementary figures and images for: Galectin-9 contributes to the pathogenesis of atopic dermatitis via T cell immunoglobulin mucin-3
Source: Front Immunol. 2022 Jul 22;13:952338. doi: 10.3389/fimmu.2022.952338 (PMC9364826; doi:10.3389/fimmu.2022.952338)

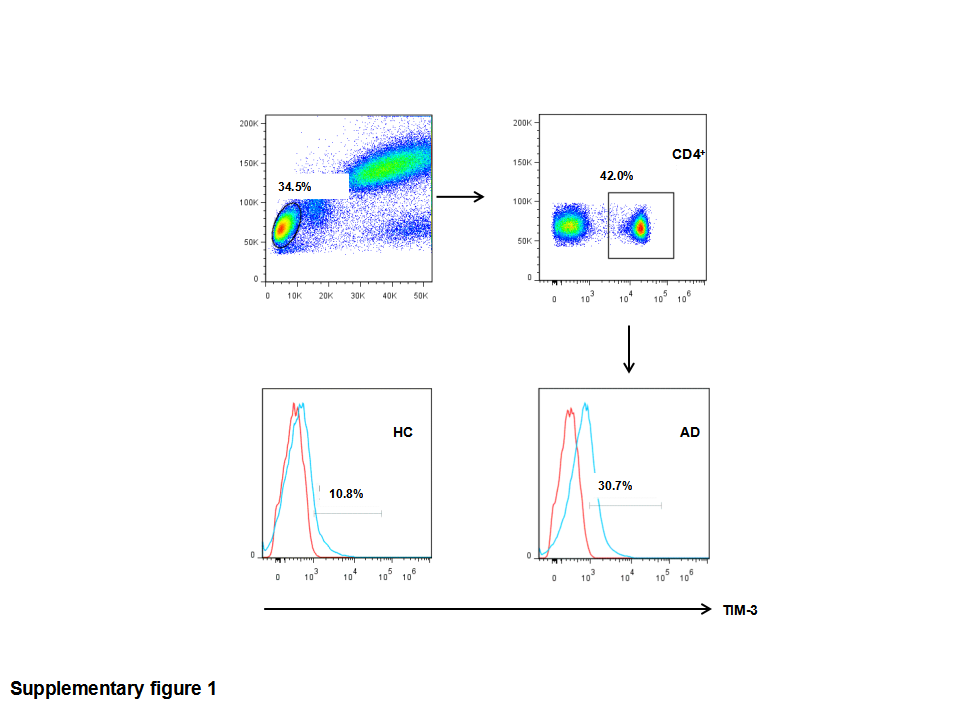

Supplement: Supplementary Figure 1 — Representative flow cytometry analysis of TIM-3 expression on CD4+T cells in whole blood of patients with AD and HC. Cells were initially gated on CD4+ T cells. Subsequently, the frequency of TIM-3+ cells CD4+ T cells was analyzed by flow cytometry in the whole blood of AD patients and HC, respectively. [file Image_1.tif]

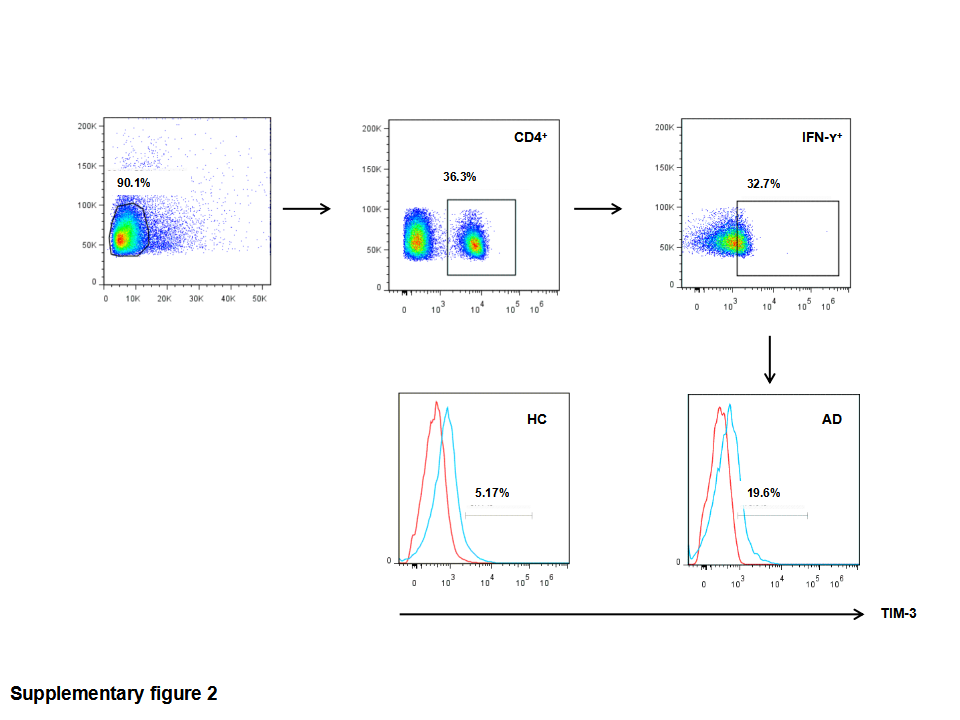

Supplement: Supplementary Figure 2 — Representative flow cytometry analysis of TIM-3 expression on CD4+IFN-γ+ T cells in PBMCs from AD patients and HC. Cells were initially gated on CD4+ T cells. Then IFN-γ+cells were selected out of the gated CD4+ T cells. Subsequently, the frequency of TIM-3+ cells on CD4+IFN-γ+ T cells was analyzed by flow cytometry in PBMCs from AD patients and HC, respectively. [file Image_2.tif]

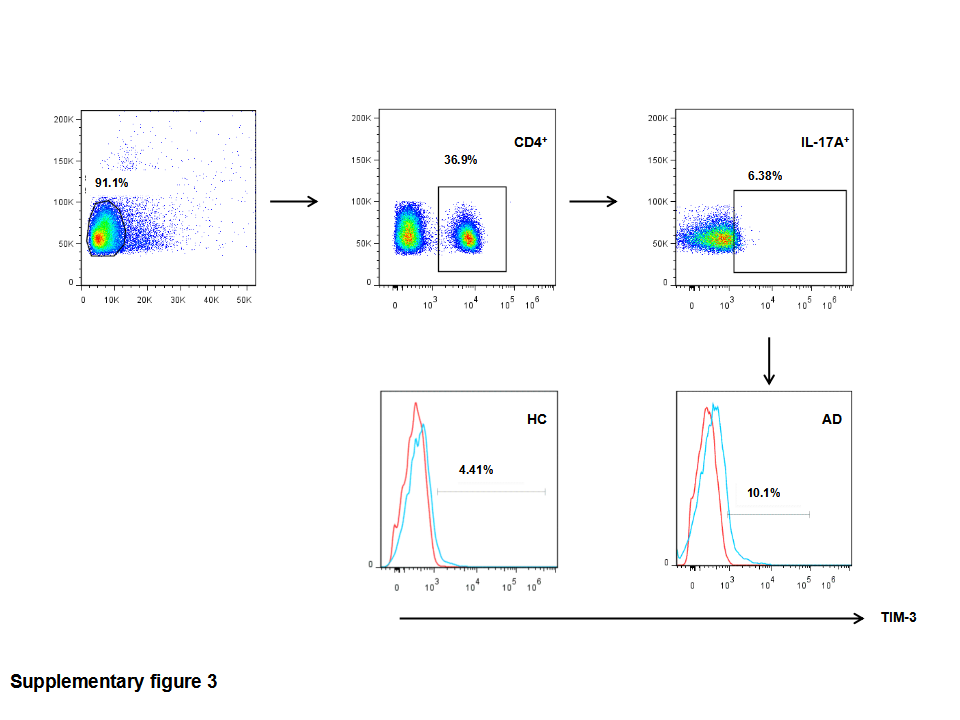

Supplement: Supplementary Figure 3 — Representative flow cytometry analysis of TIM-3 expression on CD4+IL-17A+ T cells in PBMCs from AD patients and HC. The cells were initially gated on CD4+ T cells. Then IL-17A+ cells were selected out of the gated CD4+ T cells. Subsequently, the frequency of TIM-3+ cells on CD4+IL-17A+ T cells was analyzed by flow cytometry in PBMCs from AD patients and HC, respectively. [file Image_3.tif]

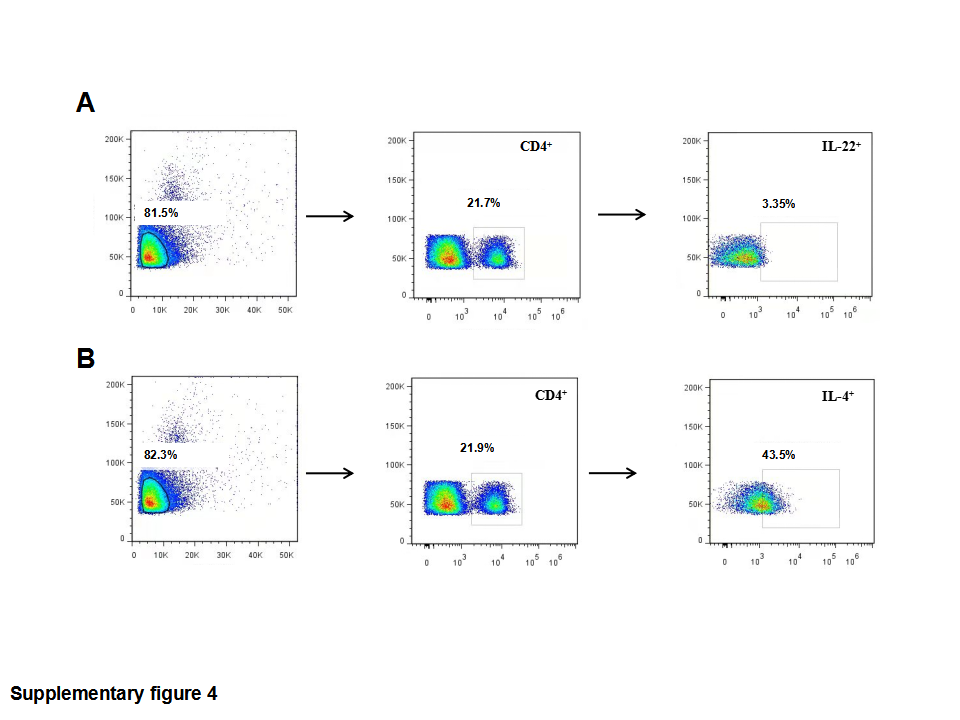

Supplement: Supplementary Figure 4 — Representative flow cytometry analysis of TH22 and TH22 in PBMCs from AD patients. (A) TH22 cells: Cells were initially gated on CD4+ T cells. Then IL-22+cells were selected out of the gated CD4+ T cells. (B) TH2: Cells were initially gated on CD4+ T cells. Then IL-4+cells were selected out of the gated CD4+ T cells. [file Image_4.tif]
